# Stoichiometric constraints modulate temperature and nutrient effects on biomass distribution and community stability

**DOI:** 10.1111/oik.08601

**Published:** 2021-10-26

**Authors:** Arnaud Sentis, Bart Haegeman, José M. Montoya

**Affiliations:** Theoretical and Experimental Ecology Station, CNRS, Moulis, France

**Keywords:** biomass structure, consumer–resource dynamics, nutrient quota, paradox of enrichment, stoichiometry, temperature, temporal variability, trophic interactions

## Abstract

Temperature and nutrients are two of the most important drivers of global change. Both can modify the elemental composition (i.e. stoichiometry) of primary producers and consumers. Yet their combined effect on the stoichiometry, dynamics and stability of ecological communities remains largely unexplored. To fill this gap, we extended the Rosenzweig–MacArthur consumer–resource model by including thermal dependencies, nutrient dynamics and stoichiometric constraints on both the primary producer and the consumer. We found that stoichiometric and nutrient conservation constraints dampen the paradox of enrichment and increased persistence at high nutrient levels. Nevertheless, stoichiometric constraints also reduced consumer persistence at extreme temperatures. Finally, we also found that stoichiometric constraints and nutrient dynamics can strongly influence biomass distribution across trophic levels by modulating consumer assimilation efficiency and resource growth rates along the environmental gradients. In the Rosenzweig–MacArthur model, consumer biomass exceeded resource biomass for most parameter values whereas, in the stoichiometric model, consumer biomass was strongly reduced and sometimes lower than resource biomass. Our findings highlight the importance of accounting for stoichiometric constraints as they can mediate the temperature and nutrient impact on the dynamics and functioning of ecological communities.

## Introduction

Temperature and nutrients regulate many biological processes, including species geographical distribution, primary production, species interactions and energy and material fluxes (Falkowski et al. 1998, Enquist et al. 1999, Elser et al. 2007, Thomas et al. 2017). They are at the core of several ecological theories. While temperature is a fundamental component of metabolic scaling theory (Brown et al. 2004), nutrients are at the core of resource competition theory (Tilman 1982) and ecological stoichiometry (i.e. the element composition of organisms) theory (Sterner and Elser 2002). Cross et al. (2015) suggested that a better understanding of the interactions between

Published by John Wiley & Sons Ltdtemperature and nutrients is crucial for developing realistic predictions about ecological responses to multiple drivers of global change, including climate warming and elevated nutrient supply. Nutrients can modulate the effects of warming on communities directly by altering primary production, and/or indirectly by changing the elemental composition of primary producers. Conversely, thermal effects on trophic interaction strengths (i.e. the per capita effect of predators on prey population densities) and on consumer energetic efficiencies (i.e. ingestion relative to metabolic demand) depend on both the quantity and quality of their resources. While Cross et al. (2015) provided a road map on how to investigate the combined effects of temperature and nutrients on ecological processes, we still lack an integrative theory to better understand how the links between stoichiometry, nutrient enrichment and temperature influence the dynamics and stability of multispecies communities. Such a theory will allow us to understand how and when stoichiometric variation modulates the consequences of single and combined components of global change on trophic interactions, community dynamics and ecosystem functioning.

Predicting the effects of global warming and nutrient changes on ecosystems is challenging as species are embedded within communities of multiple interacting species (Petchey et al. 1999, Tylianakis et al. 2008, Montoya and Raffaelli 2010, Gilbert et al. 2014). Increased resource availability (hereafter: enrichment) and warming can jointly affect food-web stability and structure by modifying the strength of trophic interactions (O’Connor et al. 2009, Binzer et al. 2012, 2016, Kratina et al. 2012, Sentis et al. 2014, Synodinos et al. 2021). Enrichment typically increases energy flux from resources to higher trophic levels which often leads to the well-known paradox of enrichment where the amplitude of population fluctuations increase with nutrients, leading to extinctions at high nutrient concentrations (Rosenzweig 1971, Rip and McCann 2011, Gilbert et al. 2014). Nevertheless, most consumer species become less efficient at processing matter and energy at warmer temperatures as their metabolic rates often increase faster with temperature than their feeding rates (Vucic-Pestic et al. 2011, Fussmann et al. 2014, Iles 2014). This reduction of energetic efficiency lessens energy flow between trophic levels and can hence stabilizes food-web dynamics by reducing population fluctuations as long as interaction strength decreases faster with warming than maximal energetic efficiency (Synodinos et al. 2021).

As a result, mild warming may alleviate the paradox of enrichment by decreasing interaction strength and consumer energetic efficiency (Binzer et al. 2012, Sentis et al. 2017, Synodinos et al. 2021).

The theoretical expectations and results described above have already improved our ability to understand and predict the effects of temperature and enrichment on food webs (Boit et al. 2012, Tabi et al. 2019). However, most previous studies using metabolic scaling theory assumed that nutrient enrichment lead to an increase in resource carrying capacity without influencing resource elemental composition (Vasseur and McCann 2005, Binzer et al. 2012, 2016, Gilbert et al. 2014, Sentis et al. 2017). Yet nutrient enrichment effects are more complex. The elemental composition of primary producers is likely to be altered, in response to the supplies of energy and materials relative to their growth and nutrient intake rates (Rastetter et al. 1997, Sterner et al. 1997, Finkel et al. 2009). This, in turn, can affect the dynamics of the producer population and the herbivores feeding on it. For instance, previous modelling studies showed that introducing stoichiometric heterogeneity in predator–prey population dynamic models can dampen the negative effect of nutrient enrichment on system persistence by reducing population biomass fluctuations (Andersen 1997, Loladze et al. 2000, Andersen et al. 2004, Elser et al. 2012). More generally, the stoichiometric flexibility of primary producers, in particular the flexibility in carbon to nutrient ratios (e.g. C:N or C:P), has important implications for animal feeding behaviour (White 1993), consumer population stability (White 1993, Sterner and Hessen 1994, Hessen et al. 2002), community structure (Andersen 1997) and ecosystem processes such as biogeochemical cycling (Andersen 1997, Hessen et al. 2004).

Previous theoretical and empirical studies reported that stoichiometric variations can have a strong influence on the stability of consumer–resource interactions (Andersen 1997, Andersen et al. 2004, Diehl et al. 2005, Elser et al. 2012). For instance, populations of crustacean *Daphnia* feeding on low quality (i.e. low nutrient: carbon ratio) algae cannot persist even when resource quantity is not a limiting factor (Elser et al. 2007). Consumer extinction is explained by the fact that the consumer assimilation efficiency is, for most organisms, a function of resource quality (Elser et al. 2000). When resource quality is low, the consumers assimilate only few nutrients relative to the biomass they ingest, which limits their growth and reproduction (Elser et al. 2000, 2012). Temporal variations in resource quality can stabilize the system by weakening interaction strength and dampening population fluctuations (Andersen et al. 2004, Diehl et al. 2005; but see Loladze et al. 2000, Elser et al. 2012). However, it remains unclear whether and how temporal variations in the elemental composition of primary producers and consumers can modulate the effects of temperature and nutrients on important community features such as stability and biomass distribution across trophic levels. Previous studies indicated that the spatial and temporal intraspecific variations in the elemental composition of primary producers are expected to increase in response to global change drivers such as temperature, CO_2_ and nutrient availability (Bezemer and Jones 1998, Woods et al. 2003, Finkel et al. 2009). This increased variation can be of importance for both primary producer and consumer populations as the growth rate of primary producers is well known to depend on their elemental composition (Droop 1974) as is the assimilation efficiency of the consumers (Sterner and Elser 2002).

Altogether, previous studies indicated that both temperature and stoichiometric variations can have important effects on species interactions and community dynamics (Andersen et al. 2004, Diehl et al. 2005, Fussmann et al. 2014, Binzer et al. 2016, Sentis et al. 2017, Synodinos et al. 2021). However, the effects of temperature and nutrient stoichiometry on food web dynamics and stability have only been studied in isolation. Recent theory by Uszko et al. (2017) showed that considering nutrient dynamics can help to better understand the influence of temperature on consumer–resource population dynamics and resource carrying capacity. Nutrient conservation (i.e. mass balance) constrains the dynamics of both the resource and consumer populations that fluctuate less than in other models not considering nutrient dynamics and conservation. Nevertheless, Uszko et al. (2017) considered that the elemental composition of both the resource and the consumer are constant and independent of temperature and nutrient dynamics. This contrasts with the empirical observation that resource elemental composition is flexible and can vary with both temperature and nutrient dynamics (Droop 1974, Elser et al. 2000, Woods et al. 2003). Here we thus focused on the combined effects of temperature and nutrients on the stoichiometry of primary producers and how this affects community stability and biomass distribution across trophic levels in a consumer–resource system. Understanding the determinants of stability and biomass distribution has been at the core of ecology for a long time (Elton 1927, Lindeman 1942). Recent theory aims at explaining empirical observations of trophic pyramids (i.e. population biomass decreases with trophic levels), inverted trophic pyramids (i.e. population biomass increases with trophic levels), trophic cascades and the link between biomass distribution and stability (McCauley et al. 2018, Barbier and Loreau 2019).

Here, we used the Rosenzweig–MacArthur model as a baseline non-stoichiometric model because this model is one of the most studied models used to investigate the effects of temperature and nutrient enrichment on community dynamics (Vasseur and McCann 2005, Binzer et al. 2012, Fussmann et al. 2014, Sentis et al. 2017, Synodinos et al. 2021). Inspired by previous temperature-independent stoichiometric consumer–resource models (Andersen 1997, Andersen et al. 2004, Diehl et al. 2005), we then extended the Rosenzweig–MacArthur model to account for nutrient dynamics with nutrient conservation, as well as for the simultaneous dependence of community dynamics on temperature and flexible resource stoichiometry. Our objective here was not to develop a complex and very realistic stoichiometric model that would include additional important abiotic and biotic features such as light intensity (Diehl 2007) or compensatory feeding (Cruz-Rivera and Hay 2000). Instead, we aimed at introducing two fundamental stoichiometric features (i.e. stoichiometric flexibility and stoichiometric imbalance) and investigate how these stoichiometric considerations can change predictions of the Rosenzweig–MacArthur model. We thus used our extended Rosenzweig–MacArthur model to predict the effects of warming and nutrient enrichment on population dynamics and biomass distribution across trophic levels and compared these predictions with the predictions of the nonstoichiometric Rosenzweig–MacArthur model. We particularly addressed two questions: 1) how do stoichiometric constraints modulate the effects of enrichment and warming on community stability and persistence? And 2) how do stoichiometric constraints modulate the effects of enrichment and warming on biomass distribution across multiple trophic levels?

## Methods: population dynamic models

### The Rosenzweig–MacArthur (RM) model

Rates of change of the consumer and resource biomass densities $\overset{\prime }{C}$ and $\overset{\prime }{R}$ depend on their respective biomass densities *C* and *R* (g m ^−3^):

(1) $$\overset{\prime }{R}=r\left(1-\frac{R}{K}\right)R-\frac{aR}{1+ahR}C$$

(2) $$\overset{\prime }{C}=\left(e\frac{aR}{1+ahR}-m\right)C$$

The population growth rate of the resource is given by the logistic equation where *r* is the resource maximum growth rate and *K* is the resource carrying capacity. The population growth rate of the consumer is equal to its feeding rate multiplied by its assimilation efficiency *e* (i.e. the fraction of resource biomass converted into consumer biomass) minus a loss term associated to metabolic losses *m*. The feeding rate of the consumer *C* depends on the density of its resource *R* and follows a Holling type II functional response, with consumer–resource attack rate *a* and handling time *h*.

In the RM model, consumer and resource population growth rates are only limited by nutrient or resource density. Nutrient enrichment is assumed to increase resource carrying capacity, which often leads to the well-known paradox of enrichment where populations fluctuates up to extinctions (Rosenzweig 1971). Nevertheless, this model neither considers nutrient dynamics nor temporal variations of resource stoichiometry and their consequences on population dynamics. Because nutrient conservation is not guaranteed in the RM model, the nutrient content of the resource and consumer populations can exceed the total amount of nutrient in the system and thus violate mass balance, which can lead to unrealistically large population fluctuations (Andersen et al. 2004). To circumvent these limitations of the RM model, we extended it to better consider nutrient dynamics (and nutrient conservation), resource stoichiometry and the way they can affect resource and consumer population dynamics.

### The stoichiometric Rosenzweig–MacArthur (SRM) model

We derived a stoichiometric extension of the Rosenzweig–MacArthur consumer–resource model with additional stoichiometric and temperature dependencies of several biological rates. We considered two stoichiometric constraints: one on the resource population growth rate, and the other on the consumer assimilation efficiency (see below for more details). These stoichiometric constraints have been observed for several consumer–resource pairs suggesting that they are core components of species growth and interactions (Sterner and Elser 2002).

#### Stoichiometric constraint on the resource population growth rate

Inspired by previous stoichiometric models (Andersen 1997, Loladze et al. 2000, Andersen et al. 2004, Diehl et al. 2005), we extended the RM model by considering explicit nutrient dynamics and nutrient effects on resource population growth rate. The system is assumed to be closed for nutrients. Thus, nutrient supply originates exclusively from biomass excretion and remineralization. We assume that free nutrients are taken up very quickly relative to the dynamics of the consumer and resource biomasses, as is often the case in aquatic systems. The total amount of nutrients in the system (*N*_tot_) is then a measure of nutrient enrichment.

In contrast to the very high plasticity in C:N or C:P exhibited by autotrophs, heterotrophs regulate elemental composition within narrower bounds, even when consuming food with large variation in elemental composition (Andersen and Hessen 1991, Sterner and Hessen 1994, Andersen 1997, Elser et al. 2000). In other words, the elemental homeostasis is much stronger for consumers compared to primary producers. We thus assumed the nutrient quota (i.e. the nutrient to carbon ratio) of the consumer *Q*_C_ to be conserved whereas the one of the resource *Q*_R_ is flexible over time with the only constraint that *Q*_R_ > *Q*_min_. As in the RM model, rates change of the consumer and resource biomass densities $\overset{\prime }{C}$ and $\overset{\prime }{R}$ depend on their respective carbon biomass densities *C* and *R* (gC m^−3^), except that the resource population growth rate follows the Droop equation (Droop 1974) given by *r*(1 – *Q*_min_/*Q*_R_)*R* and is now limited by *Q*_R_ relative to the minimum nutrient quota *Q*_min_:

(3) $$N_{\text{tot }}=Q_{\text{R}}R+Q_{\text{C}}C$$

(4) $$\overset{\prime }{R}=r\left(1-\frac{Q_{\min}}{Q_{\text{R}}}\right)R-\frac{aR}{1+ahR}C$$

(5) $$\overset{\prime }{C}=\left(e\frac{aR}{1+ahR}-m\right)C$$

From the nutrient conservation equation (Eq. 3) we obtain that $Q_{\text{R}}=\frac{N_{\text{tot}}-Q_{\text{C}}C}{R}$. The intuitive interpretation is that the resource nutrient quota *Q*_R_ changes instantaneously with the density of the resource population *R* and with the concentration of the nutrient stored in the consumer biomass *Q*_C_*C*, to maintain nutrient balance (details in the Supporting information). This contrasts with the RM model where nutrient conservation is not guaranteed and where the sum of the resource and consumer population’s nutrient content can exceed the total amount of nutrient in the system.

#### Stoichiometric constraint on the consumer population growth rate

In the RM model, the growth rate of the consumer population only depends on resource density. In other words, the RM model assumes that resource stoichiometry is not limiting and conversion efficiency *e* is often taken for a consumer feeding on a high quality resource (Yodzis and Innes 1992, Binzer et al. 2012, Fussmann et al. 2014, Uszko et al. 2017). However, conversion efficiency can be much lower when the resource is of poor quality (i.e. when there is a stoichiometric unbalance between the consumer and the resource nutrient: carbon ratio) (Elser et al. 2000, 2007). We relaxed this assumption of the RM model by making the population growth rate of the consumer dependent on both resource quality (i.e. nutrient quota) and quantity (i.e. biomass density). In the SRM model, consumer production is also limited by resource quality as the consumer assimilation efficiency *e* is a saturating function of resource nutrient quota *Q*_R_:

(6) $$e\left(Q_{\text{R}}\right)=e_{\max}\frac{Q_{\text{R}}}{Q_{\text{R}}+Q_{\text{C}}}$$

The intuitive interpretation of Eq. 6 is that resource quality is not a limiting factor for consumer growth as long as the nutrient content of the resource is superior to the nutrient content of the consumer (i.e. *Q*_R_ > *Q*_C_). In other words, *e*(*Q*_R_) is proportional to *Q*_R_ for *Q*_R_ < *Q*_C_ and is at its maximum (*e*_max_) for *Q*_R_ >> *Q*_C_. The later scenario corresponds to the assumption of the RM model where conversion efficiency is taken for a high-quality resource and thus *e* = *e*_max_. By replacing *e* by *e*(*Q*_R_) in Eq. 5, we obtain the SRM model.

#### Temperature dependence of model parameters

To investigate the effect of temperature and stoichiometric constraints on consumer–resource dynamics, we next extended the RM and SRM models described above by adding thermal dependencies of the parameters. Following Uszko et al. (2017), we assumed that the total amount of nutrient *N*_tot_, the maximum food conversion efficiency *e*_max_ and fixed stoichiometric traits (*Q*_C_) are independent of temperature, as there is no evidence of systematic temperature dependence for any of them (Peters 1983, Ahlgren 1987, Borer et al. 2013, Yvon-Durocher et al. 2015). Rate of maintenance respiration and natural background mortality *m* typically increases exponentially with temperature (Supporting information). We thus used the Arrhenius equation to describe the effect of temperature *T* (in Kelvin) on *m*:

(7) $$m(T)=m_{0}e^{\frac{-E_{m}}{kT}}$$

where *m*_0_ is a parameter-specific constant calculated at temperature of 0°C (= 273.15 K). The temperature dependence is characterized by the respective activation energy *E*_m_ (eV) and the Boltzmann constant *k* = 8.62 × 10^−5^ eV K^−1^. As the temperature dependencies of resource intrinsic growth rate *r* and functional response parameters (*a*, 1/*h*) are often unimodal rather than exponential (Englund et al. 2011, Rall et al. 2012, Sentis et al. 2012, Thomas et al. 2012, Synodinos et al. 2021), we used Gaussian functions for *r* and *a* and an inverted Gaussian function for *h*:

(8) $$g(T)=g_{0}e^{\pm \frac{{\left(T-T_{\text{opt}}\right)}^{2}}{2s^{2}}}$$

where *T*_opt_ is the temperature at which the rate *g* reaches its minimum or maximum, *s* is the function width and *g*_0_ is a parameter-specific constant calculated at *T*_opt_. The minussign corresponds to Gaussian functions and the plus-sign to inverted Gaussian functions.

### Model parameterisation and simulations

To parameterise the models we assumed the resource and consumer species to be a unicellular freshwater algae and a *Daphnia* grazer, respectively. The choice for this system was motivated by the good characterization of both the stoichiometric parameters and thermal dependencies for this system (Andersen 1997, Uszko et al. 2017). Uszko et al. (2017) recently estimated the thermal dependencies for biological rates of the green algae *Monoraphidium minutum* and the grazer *Daphnia hyalina*. We thus used their estimates of stoichiometric parameters and thermal dependencies (see the Supporting information for further details).

To investigate the individual and combined effects of enrichment, warming and stoichiometric constraints, we varied temperature (401 values ranging from 0 to 40°C by 0.1°C) and total amount of nutrients (parameter *N*_tot_ in Eq. 10; 60 values ranging from 0.001 to 0.06 gP m^−3^ by 0.001 gP m^−3^, overlapping with reported mean phosphorus concentration in European peri-alpine lakes (Anneville et al. 2005)). For the RM model, we used the minimum nutrient quota to convert nutrients into resource (i.e. *K* = *N*_tot_/*Q*_min_). This implies that carrying capacity is independent of temperature which is expected for closed, nutrient-limited systems (Uszko et al. 2017) although more experimental evidence are needed to verify this assumption (but see Bernhardt et al. 2018). We then simulated the consumer–resource dynamics for 1000 days to enable the system to reach an attractor (either an equilibrium point or a limit cycle) before we assessed the final state. Therefore, for each model, we simulated 24 060 combinations of environmental conditions (401 temperatures by 60 nutrient concentrations). Initial biomass density of each species was set to 0.98 times its equilibrium density in the two-species system (calculated by solving for the two-species equilibrium, using either Eq. 1–2 for model RM or Eq. 3–5 for model SRM). The value of 0.98 was chosen to be 1) close enough to equilibria to avoid extinctions caused solely by transient dynamics and 2) not exactly the equilibrium value to probe the stability of the equilibrium. Additionally, at the end of each simulation, we used the last 100 time steps to calculate to coefficient of variation CV (standard deviation divided by mean population density) of each species to assess if populations were fluctuating or at equilibrium (CV is zero when the system is at equilibrium and positive when populations fluctuate). Any population falling below the extinction threshold of 10^−9^ g m^−3^ during the simulations was deemed extinct and its biomass set to zero to exclude ecologically unrealistic low biomass densities. For each model, we calculated system persistence as the percentage of simulations with the two species remaining extant at the end of the simulations. We also calculated system persistence without considering the extinction threshold to assess the proportion of extinctions that are driven by population fluctuations resulting in unrealistic low biomass densities.

To reveal the dynamic effects of the stoichiometric constraints, we calculated the values of assimilation efficiencies and carrying capacities predicted by the SRM model for each temperature–nutrient scenario and used these effective parameter values to replace the values of parameters *e* and *K* in the RM model for each temperature–nutrient scenario. The objective of using these effective parameter values was to disentangle the static effect of stoichiometric constraints (i.e. changing the average parameter values of consumer assimilation efficiency and of the resource carrying capacity) from their population dynamical effects. Population dynamics were simulated with R ver. 3.4.3 (<www.r-project.org>) using the ‘deSolve’ package (Soetaert et al. 2012) with an absolute error tolerance of 10^−10^ and a relative error tolerance of 10^−6^.

## Results

### Stability: population fluctuations and persistence

Considering stoichiometric constraints and nutrient dynamics dampened the paradox of enrichment, reducing fluctuations at high nutrient levels and hence increasing persistence. However, the persistence of the consumer at low and high temperatures was reduced in the SRM model compared to the RM model. As a result, the overall effect of stoichiometric constraints and nutrient dynamics on stability depends on their relative influence on population fluctuations versus consumer persistence. In the two following paragraphs, we explain in more detail these results and highlight key differences between the outcomes from RM and SRM models.

**Figure 1:**
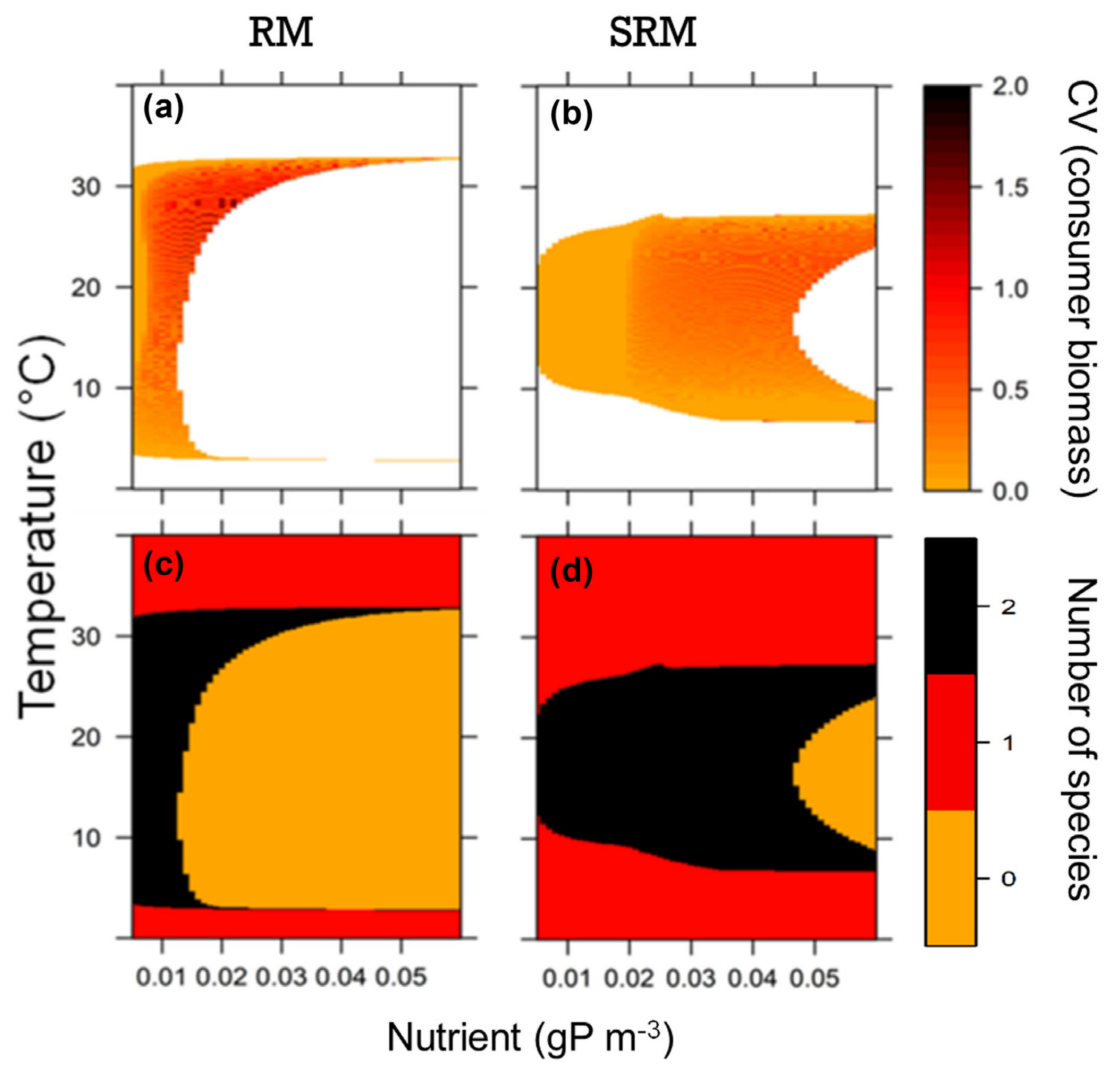
Population fluctuations (consumer biomass coefficient of variation (a) and (b)) and species persistence (number of species; (c) and (d)) across the temperature (y-axis) and nutrient (x-axis) gradients as predicted by the Rosenzweig–MacArthur (RM; (a) and (c)) and by the stoichiometric Rosenzweig–MacArthur (SRM; (b) and (d)) models. In (a) and (b), coefficient of variation (hereafter CV) represents fluctuation amplitudes. CV is null when the system is at equilibrium and positive when populations fluctuate. In (a) and (b), the white colour corresponds to the temperature–nutrient scenario for which the consumer has gone extinct whereas the orange to red to dark red represent population fluctuations of increasing amplitude. In (c) and (d), in black: both consumer and resource persist; in red: only the resource persists; in orange: none persists. Resource biomass CV is not shown; it is qualitatively similar to the consumer biomass CV as resource and consumer biomass fluctuation are strongly coupled.

The RM model predicts that increasing nutrient concentration is strongly destabilizing: the system shifts from a stable equilibrium point to limit cycles (i.e. the system crosses a Hopf bifurcation, Fig. 1: consumer CV goes from zero to positive values when nutrient concentration increases). This destabilizing effect is known as the paradox of enrichment (Rosenzweig 1971). As population biomass fluctuations (i.e. cycle amplitude, represented by increasing CV values in Fig. 1a) increase with nutrient concentration, minimal population densities are very low at high nutrient concentrations leading to the extinction of the resource once the extinction threshold is crossed and then the consumer goes to extinction as it cannot survive without resource (Fig. 1a). In the range of temperatures where the consumer persists, warming does not have a strong influence on the nutrient concentration at which the system shifts from the stable equilibrium point to limit cycles (CV > 0 in Fig. 1), although this qualitative shift is absent at very high temperatures (i.e. 32°C) when the consumer is close to extinction. Warming enhances the persistence of the consumer–resource system at high nutrient concentrations (Fig. 1c). This is explain by the fact that, at low temperatures, when the system starts fluctuating with increasing nutrient concentration, the fluctuations are so large that they systematically lead to extinction (Fig. 1a). At higher temperatures, the system fluctuates but the fluctuation amplitudes are smaller which thus dampens extinctions driven by the paradox of enrichment. However, very warm and cold temperatures cause the extinction of the consumer (see below for the mechanisms underlying extinctions), releasing resources from top to down control. Overall, we found that, without considering the extinction threshold of 10^−9^ g m^−3^, both the consumer and the resource can persist in 74% of the temperature–nutrient concentration scenarios (i.e. black + orange areas in Fig. 1c). Nevertheless, when considering the extinction threshold, they persist in only 21% of the temperature–nutrient scenarios (i.e. black area in Fig. 1c). In other words, comparing the model simulations with and without extinction threshold revealed that, in the RM model, extinctions are mostly driven by population fluctuations leading to very low biomass densities at which the population is at risk of extinction.

In contrast, the SRM model shows that increasing nutrient concentrations causes fewer fluctuations than those observed for the RM model (Fig. 1b, d). This is because: 1) more nutrients are needed to shift the system from a stable equilibrium point to limit cycles the system can indeed persist without fluctuations (i.e. CV = 0) up to 0.02 gP m^−3^ whereas it was only up to 0.0005 gP m^−3^ in the RM model – and 2) when the system fluctuates (i.e. CV > 0), the amplitude of the fluctuations is smaller in the SRM than in the RM model. As a result, stoichiometric and nutrient conservation constraints dampen the amplitude of population fluctuations (i.e. the paradox of enrichment) and hence increase system persistence at high nutrient levels. While the qualitative effect of temperature is similar to that observed in the RM model, the thermal thresholds for consumer persistence are reduced at low and high temperatures in the SRM predictions (Fig. 1b, d). Moreover, thermal thresholds remain almost constant along the nutrient gradient in the RM model, whereas in the SRM model they depend on nutrient concentration, with a smaller thermal range at low nutrient levels compared to high nutrient levels (Fig. 1b, d). The consumer is thus more likely to go extinct at low nutrient concentrations and extreme temperatures in the SRM model than in the RM model. Overall, system persistence for the SRM model was 44% without considering the extinction threshold and 37% when considering it. In other words, comparing the model simulations with and without extinction threshold revealed that, in the SRM model, few extinctions are driven by population fluctuations leading to very low biomass densities. We thus conclude that the RM model predicts larger population fluctuations leading to high probabilities of populations extinctions in comparison to the SRM model.

### Biomass distribution

**Figure 2:**
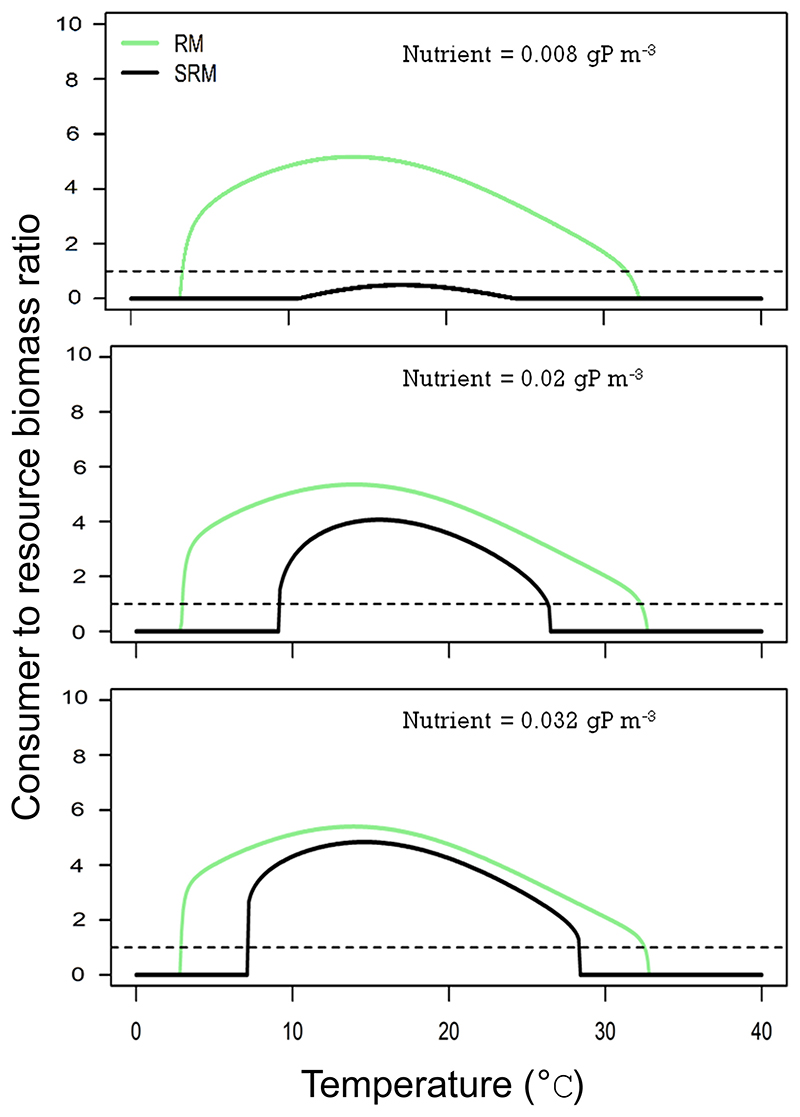
Consumer–resource biomass ratio along the temperature gradient for the Rosenzweig–MacArthur (RM, green lines) and the stoichiometric Rosenzweig–MacArthur (SRM, black lines) models at three nutrient concentrations (0.008, 0.02 and 0.032 gP m^−3^). In each panel, the dotted line represents biomass ratio of one; i.e. the biomass densities of the resource and the consumer are equal. Biomass values shown at equilibrium points. For unstable equilibrium points (i.e. limit cycles), see the Supporting information.

We next compared the predictions of both models for consumer–resource biomass ratios along the temperature and nutrient gradients (Fig. 2). We found that the RM model systematically predicts biomass ratios > 1 (i.e. consumer biomass is larger than resource biomass). In contrast, the SRM model predicts biomass ratios both > or < than 1 depending on temperature and nutrient levels. The RM model predicts that, as soon as the consumer can persist, its population biomass density always exceeds the resource population biomass density (Fig. 2). With the SRM model, the biomass ratios are below one at low nutrient levels (Fig. 2). However, at medium and high nutrient levels, the ratios are above one as soon as the consumer can persist. We found qualitatively similar results when considering unstable equilibrium points (Supporting information). Finally, we showed that, for equivalent parameter values, the RM model predicts biomass ratio that are superior or equal to the ones predicted by the SRM model (Supporting information). This difference between the two models is independent of the shape and position of the temperature function used to parametrise the models.

### Mechanisms underlying stability and biomass distribution patterns

Here, we detail the mechanisms underlying the stability and biomass distribution patterns to better understand how and when stoichiometric constraints modulate the effects of temperature and nutrients on consumer–resource dynamics. The first mechanism corresponds to the effect of stoichiometric constraints on the consumer energetic efficiency that determines the consumer persistence at extreme low and high temperatures. The second mechanism relates to the influence of the stoichiometric constraints on population dynamical feedback that explains why the stoichiometric model predicts more stability at high nutrient levels compared to the non-stoichiometric model.

### Consumer energetic efficiency

The persistence of the consumer at low and high temperatures is driven by the energetic efficiency *EE* of the consumer (i.e. its feeding rate relative to metabolic losses) calculated as follows:

(9) $$EE=\frac{ef\left(R^{*}\right)}{m}$$

where *f*(R*) is the functional response of the consumer at resource density R* (i.e. the resource equilibrium density in absence of the consumer). We recall that the assimilation efficiency *e* is a function of resource quality *Q*_R_ in the SRM model whereas it is assumed constant in the RM model. The intuitive interpretation of Eq. 9 is that *EE* should be above one for the consumer population to grow and persist. *EE* is equivalent to the invasion rate of the consumer into a system with resource only.

**Figure 3:**
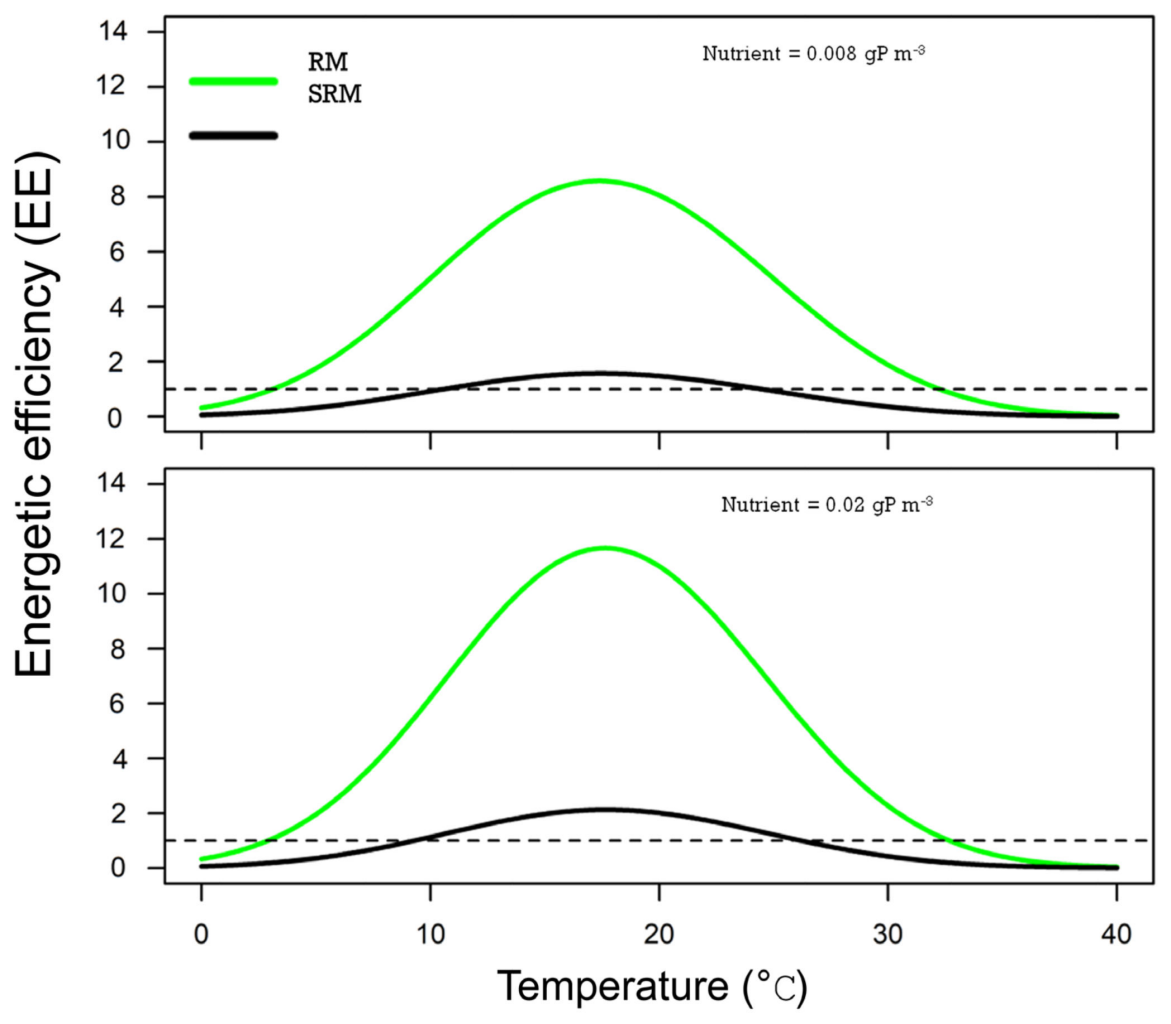
Consumer energetic efficiency along the temperature gradient for the Rosenzweig–MacArthur (RM, in green) and the stoichiometric Rosenzweig–MacArthur (SRM, in black) models at two nutrient concentrations (0.008 and 0.02 gP m^−3^). In each panel, the dotted line represents energetic efficiency equal to one.

To better understand the influence of stoichiometric constraints on consumer persistence, we thus investigated differences in the RM and SRM model predictions regarding the consumer energetic efficiency *EE* along the temperature gradient at two nutrient concentrations (Fig. 3). For both models and the parameter values we used, energetic efficiency at equilibrium has a hump-shaped relationship with temperature with maximal efficiency values at medium temperatures. While this unimodal shape is conserved across nutrient levels and models, the RM model systematically predicts higher consumer energetic efficiency values than the SRM model because consumer assimilation efficiency is lower in the SRM than in the RM model (Supporting information). As a result, the temperatures at which energetic efficiency falls below one and drives consumers extinct are more extreme in the RM model compared to the SRM model (Fig. 3). In other words, energetic efficiency is above one for a narrower thermal range in the SRM model. Moreover, energetic efficiency remains just above one for most temperatures at low nutrient levels which might suggest greater sensitivity to perturbations affecting consumer feeding gains or metabolic losses.

### Dynamical feedbacks due to the stoichiometric constraints

The second mechanism by which stoichiometric constraints influence consumer–resource stability and biomass distribution are the dynamical feedbacks due to stoichiometric and nutrient conservation constraints on the resource population growth rate and on the consumer energetic efficiency. In the SRM model, the growth rate of the resource population depends on both the total nutrient load and the consumer population density as *Q*_R_ = (*N*_tot_ – *Q*_C_*C*)/*R*. In other words, when consumer population increases, this decreases resource population growth by reducing both resource density (through predation) and quality (through nutrient mass balance) leading to a negative feedback on consumer population growth rate imposed by both nutrient conservation and flexible stoichiometry. In contrast, for the RM model, the negative consumer feedback is only driven by the reduction in resource density as resource quality and nutrient dynamics are not considered. In addition to this first dynamical feedback, there is a second dynamical feedback as the consumer population growth rate also depends on *Q*_R_ and thus on its own biomass density. Thus, also this second negative feedback loop limits the consumer population growth rate when its density increases. Altogether, dynamical feedbacks reduce strongly the amplitude of population fluctuations, which in turn increases resource and consumer persistence.

**Figure 4:**
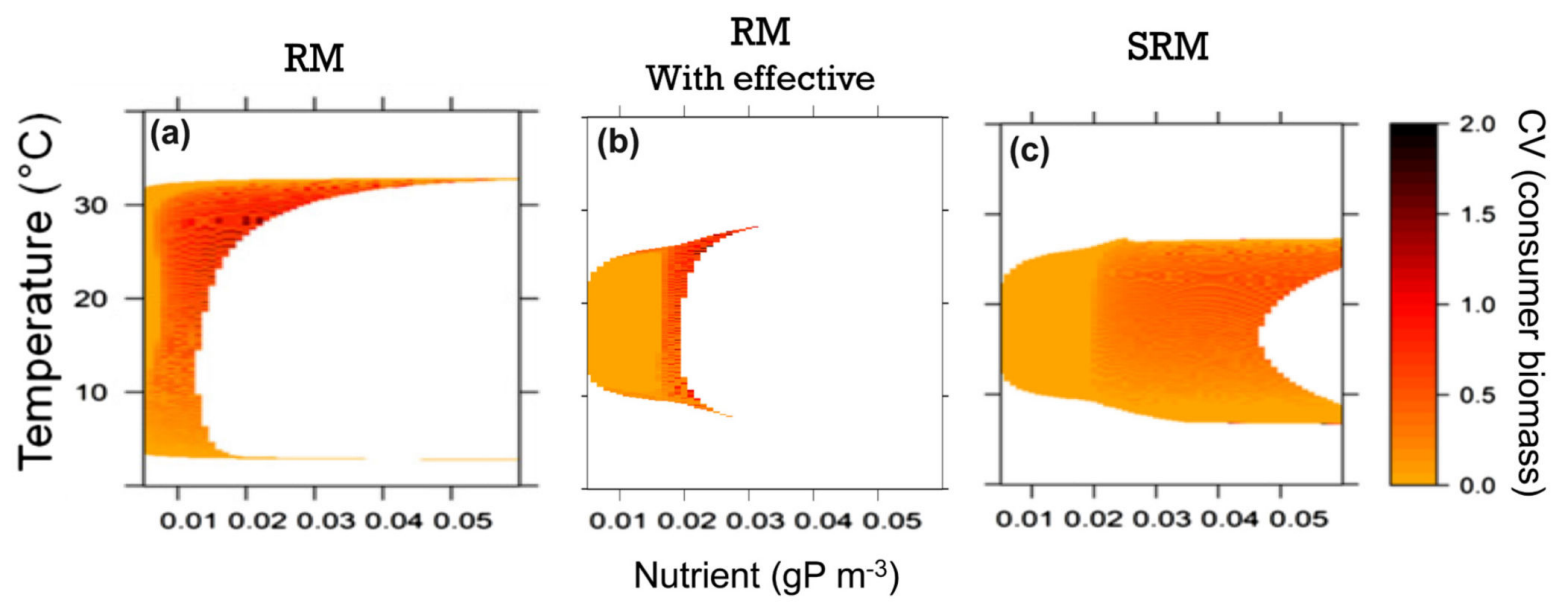
Population fluctuations (consumer biomass coefficient of variation) across the temperature (y-axis) and nutrient (x-axis) gradients as predicted by the Rosenzweig–MacArthur (RM; (a)), the RM with effective parameters (b) and the stoichiometric Rosenzweig–MacArthur (SRM; (c)) models.

To reveal the dynamic effects of the stoichiometric constraints, we calculated the values of assimilation efficiencies and carrying capacities predicted by the SRM model for each temperature–nutrient scenario (Supporting information) and used these effective parameter values to replace the values of parameters *e* and *K* in the RM model for each temperature–nutrient scenario. In other words, we calculated average values of *e* and *K* in the dynamic SRM model and used them as constant input parameters in the RM model. The objective of using these effective parameter values was to disentangle the static effect of stoichiometric constraints (i.e. changing the average parameter values of consumer assimilation efficiency and of the resource carrying capacity) from their population dynamical effect (i.e. the two dynamical feedback described above). We thus simulated population dynamics along the temperature–nutrient gradient using the RM model with these effective parameters; referred hereafter as effective RM model (Fig. 4). Comparing predictions from the RM, effective RM and SRM models allowed to disentangle the static stoichiometric effects when going from the RM to the effective RM predictions (Fig. 4a–b) from the dynamical stoichiometric effects when going from the effective RM to the SRM predictions (Fig. 4b–c). In other words, the RM and effective RM only differ in their parameter values because the effective RM takes into account the effect of stoichiometric constraints on the average parameter values. On the other hand, the effective RM and SRM have similar parameter values but different population dynamics, which helps understanding the dynamical feedback induced by stoichiometric constraints.

We found that, at low nutrient concentrations, population fluctuations and consumer persistence predicted by the effective RM model agreed with predictions of the SRM model. However, the system shifted from a stable equilibrium point to a limit cycle at lower nutrient concentrations for the effective RM model than for the SRM model. This suggests that more nutrients are needed to destabilize the system with the SRM model. Moreover, the effective RM model predicts ampler population fluctuations than the SRM model. As a result, the effective RM predicts high extinction rates at high nutrient concentrations compared to the SRM model. Overall, we found that the effective RM model cannot fully reproduce the dynamics predicted by the SRM, which indicates that including stoichiometric constraints in the RM model involves more than only changing parameter values.

## Discussion

Temperature and nutrient enrichment are two of the most important drivers of global change (Nelson 2005). However, most research on the effects of temperature and nutrients on community dynamics assumes that the elemental composition of primary producers and consumers are constant and independent of changes on energy and material fluxes (Binzer et al. 2012, 2016, Boit et al. 2012, Amarasekare and Coutinho 2014, Gilbert et al. 2014, Amarasekare 2015, Gilarranz et al. 2016, Synodinos et al. 2021). Yet, the elemental composition of primary producers is known to be flexible, which can have important consequences for community dynamics and ecosystem processes (Elser et al. 2000). We have shown how stoichiometric constraints that account for flexible stoichiometry can affect predictions on how temperature and nutrients influence community stability and biomass distribution across trophic levels. We thus argue that considering stoichiometric constraints is an important step toward a better understanding of the effects of global change on ecosystems.

### Stoichiometric constraints and temperature can dampen the paradox of enrichment

We showed that both stoichiometric constraints and temperature dampen the negative effect of nutrient enrichment on consumer–resource fluctuations and increase system persistence at high nutrient levels. Temperature effects are driven by physiological mechanisms. In agreement with previous empirical studies, our model parametrization reflects the observation that metabolic loss rates increase faster with warming than consumer feeding rates (Vucic-Pestic et al. 2011, Sentis et al. 2012, Fussmann et al. 2014, Iles 2014). Consumers are thereby less energetically efficient at higher temperatures which stabilizes food-web dynamics as long as interaction strength decreases faster with warming than maximal energetic efficiency (Synodinos et al. 2021). In contrast, as reported for previous stoichiometric models (Andersen 1997, Loladze et al. 2000, Andersen et al. 2004, Diehl et al. 2005), the effect of stoichiometric constraints is mainly linked to two mechanisms: a shift in the position of the Hopf bifurcation and negative dynamical feedbacks of the consumer and resource on their population growth rates. Both resources and consumers are composed of the same essential elements (N, P and C), which implies that the sum of essential elements contained in the resource and consumer biomasses cannot exceed the total amount of essential elements present in the system (i.e. mass balance is satisfied). As a result, when consumer or resource population biomass increases, it reduces the pool of free nutrients available for the growth of the resource population and thus limits large population fluctuations (as shown in Andersen et al. 2004). Therefore, more nutrients are needed to shift the system from a stable equilibrium to population cycles. In other words, as reported in previous theoretical studies (Andersen 1997, Loladze et al. 2000, Andersen et al. 2004, Diehl et al. 2005), the paradox of enrichment is displaced to higher nutrient concentrations (i.e. the position of the Hopf bifurcation is shifted to higher nutrient levels. In contrast, the RM model does not take into account the storage of nutrients in both the resource and consumer biomasses (i.e. the carrying capacity only depends on the total nutrient load). Mass balance is not guaranteed which implies that consumer and resource populations can reach high equilibrium biomasses and fluctuate strongly as they are not limited by nutrient conservation. Less enrichment is thus required to shift the system from a stable equilibrium point to limit cycles. Our findings are similar to previous modelling studies comparing stoichiometric and non-stoichiometric models (Andersen 1997, Loladze et al. 2000, Andersen et al. 2004, Diehl et al. 2005), and reinforce the call to better account for nutrient conservation and stoichiometry when addressing the impact of nutrient enrichment on population dynamics.

We found two dynamic effects that correspond to negative dynamical feedbacks of the consumer and the resource on themselves. The first one is imposed by nutrient conservation: when consumer population increases, it decreases the population growth rate of the resource by limiting nutrient availability, diminishing resource biomass, which, in turn, decreases the consumer population growth rate. Conversely, when the resource biomass increases, this decreases the nutrient content of the resource, which, in turn, limits the growth rates of both the resource and consumer populations. These stoichiometric negative feedback loops strongly decrease the amplitude of population fluctuations and thus dampen the paradox of enrichment. Interestingly, our comparisons of the RM, effective RM and SRM model predictions indicate that the dynamical effects contribute more to the reduction of fluctuations than the static effects: population fluctuations are large in the effective RM model accounting for the static effect only, whereas they are much smaller in SRM model accounting for both static and dynamical effects (Fig. 4). This implies that the impact of stoichiometric constraints on community dynamics goes beyond a simple modification of parameter values and encompass more complex population feedbacks between the consumer and the resource. To disentangle the relative contribution of flexible stoichiometry versus nutrient dynamics for population fluctuations, it would be interesting to compare the RM and the SRM models to a model where both resource and consumer stoichiometry are fixed but where nutrient dynamics are considered and mass balance is guaranteed (Uszko et al. 2017).

Overall, these results demonstrate that considering both flexible stoichiometry and temperature can synergistically dampen the paradox of enrichment by two different mechanisms: population dynamic feedbacks and physiological constraints. Our consumer–resource model is simplified compared to natural communities composed of numerous species. Moreover, in natural systems, a large amount of nutrient can be stored in abiotic and slow biotic pools that have long turnover times which, in turn, can influence the population dynamics. In particular, the amplitude of the population fluctuations is expected to be smaller as abiotic pools can buffer the population feedback. Nevertheless, considering the nutrient held in slow abiotic or biotic pools would not change the equilibrium densities of primary producers and grazer if nutrients are released in the environment proportionally to their concentration stored in the abiotic pool (Menge et al. 2012). Moreover, the predictions of the stoichiometric model fit with empirical observations. In eutrophic lakes and experimental mesocosms, populations can persist at relatively high nutrient concentrations even if fertilisation enhance population fluctuations (O’Connor et al. 2009, Boit et al. 2012, Kratina et al. 2012), as our stoichiometric model predicts. In contrast, the Rosenzweig–MacArthur model does not guarantee mass balance and tends to produce very large population fluctuations and extinctions at low nutrient concentrations which can explain why these predictions are not well supported by empirical observations (McAllister et al. 1972, Jensen and Ginzburg 2005).

### Effects of stoichiometric and nutrient constraints on system persistence across environmental gradients

While stoichiometric and nutrient conservation constraints dampen the paradox of enrichment and thus increase persistence at high nutrient levels, they also reduce the persistence of the consumer at low and high temperatures. Stoichiometric constraints affect the thermal thresholds for consumer extinctions. Consumers can only persist over a narrower range of intermediate temperatures when they are constrained by stoichiometry. This is due to the reduced biomass assimilation of the consumer at low and high temperatures that, in turn, decreases its energetic efficiency and thus fastens consumer extinction. In our stoichiometric model, the reduction of biomass assimilation efficiency emerges from the effect of temperature on resource quality: extreme high and low temperatures decrease resource quality and thus less resource biomass can be converted in consumer biomass at these temperatures. The emergence of a thermal dependency for assimilation efficiency contrasts with previous theoretical studies that used the RM model and assumed that the assimilation efficiency is temperature independent as resource quality is assumed constant (Binzer et al. 2012, Gilbert et al. 2014, Sentis et al. 2017, Uszko et al. 2017). In the SRM model, the thermal dependency of the consumer assimilation efficiency is fully driven by the change in the resource stoichiometry induced by temperature. The SRM model thus predicts an additional mechanism by which temperature can influence trophic interactions: temperature changes resource stoichiometry, which, in turn, impacts the consumer assimilation efficiency and its population growth rate. This prediction matches with empirical results showing that primary producer stoichiometric composition can change with temperature (Woods et al. 2003) and that consumer assimilation efficiency is sensitive to resource stoichiometric composition (Andersen 1997, Elser et al. 2000). To sum up, the overall effect of stoichiometric and nutrient conservation constraints on system persistence thus depends on the temperature range considered and on their relative influence on population fluctuations versus consumer persistence.

### Effects of stoichiometric constraints on biomass distribution

We found that stoichiometric constraints can modulate the effects of temperature and nutrients on biomass distribution across trophic levels. In the Rosenzweig–MacArthur model, biomass ratios are above one for almost all temperatures or nutrient levels as the biomass produced by the resource is efficiently transferred to the consumer level consistently along the environmental gradients. This finding agrees with theoretical studies reporting that Lotka–Volterra and RM models predict biomass ratios above one and fail to reproduce biomass pyramids for a substantial region of parameter values (Jonsson 2017, Barbier and Loreau 2019). However, in nature, consumer–resource biomass ratios are often below one (McCauley and Kalff 1981, Del Giorgio and Gasol 1995, McCauley et al. 1999, Irigoien et al. 2004) suggesting that additional mechanisms should be included to better understand and predict biomass distribution patterns in natural food webs. Our stoichiometric model agrees with experimental observations. It predicts that, at low nutrient concentrations (i.e. < 0.01 gP m^−3^), the biomass ratio never exceeds one along the entire temperature gradient. This is observed in oligotrophic aquatic systems where primary production is too low to sustain high consumer populations (O’Connor et al. 2009). In addition, we also found that increasing nutrient levels decreased the temperature ranges within which biomass ratio is below one. This corresponds to results from manipulated nutrient concentrations and temperature in aquatic mesocosms, where zooplankton to phytoplankton biomass ratio only exceeds one in the enriched mesocosms at medium or warm temperatures (i.e. 27°C) (O’Connor et al. 2009). This suggests that the models with stoichiometric constraints and nutrient conservation better reproduce the biomass patterns observed in experimental and natural systems. Nevertheless, further experiments investigating the links between stoichiometric flexibility and consumer–resource dynamics are needed to determine if these stoichiometric mechanisms are underlying patterns of biomass distribution in nature.

### Implications of our findings for global change

Temperature and nutrients do not act in isolation from each other. Climate warming, for example, causes stronger water stratification, which, in turn, can limit nutrient cycling (Sarmiento et al. 2004, Tranvik et al. 2009). Environmental policies such as the European water framework directive (i.e. Directive 2000/60/EC of the European Parliament and of the Council establishing a framework for the Community action in the field of water policy) effectively reduces input of nutrients in aquatic ecosystems (Anneville et al. 2005) while the climate keeps warming. With these two phenomena, water will often be warmer and contain fewer nutrients in aquatic systems. Our models consistently predict that warmer temperatures should stabilise consumer–resource dynamics but, if temperature further increases, the consumer goes extinct as energetic efficiency decreases with warming. Moreover, we found that stoichiometric constraints can reduce this thermal extinction threshold (i.e. the consumer persists in a narrower thermal range), especially at low nutrient levels. Our stoichiometric model thus suggests that decreasing nutrient concentrations alongside warmer temperatures should fasten the extinction of consumer populations. This prediction matches empirical observations of consumer extinctions at warm temperatures in oligotrophic aquatic systems (Petchey et al. 1999, O’Connor et al. 2009). Altogether, these results indicate that considering stoichiometric constraints can be of importance for the management of nutrient inputs and the conservation of natural populations and communities under climate change.

Knowledge of how temperature and nutrient simultaneously influence the elemental composition of primary producers and consumers is crucial to better understand and predict the effects of global change on species interactions, community dynamics and fluxes of energy and material within and among ecosystems. Here we showed that stoichiometric and nutrient conservation constraints dampen the negative effect of enrichment on stability by reducing population fluctuations through population dynamics feedbacks. However, stoichiometric constraints also decrease consumer energetic efficiency, which increases consumer extinction risk at extreme temperatures and low nutrient concentrations. Finally, stoichiometric constraints can reverse biomass distribution across trophic levels by modulating consumer efficiency and resource population growth rate along the temperature and nutrient gradients. Overall, our study suggests that accounting for stoichiometric constraints can strongly influence our understanding of how global change drivers impact important features of ecological communities such as stability and biomass distribution patterns. It opens new perspectives and possible crossing-over with prior studies. For instance, it would be interesting to assess the effects of other stoichiometric mechanisms (e.g. changes in the element limiting growth (Daufresne and Loreau 2001), the role of multiple limiting nutrients (Cherif and Loreau 2010) or of the microbial decomposer loop (Cherif and Loreau 2009)) on food web stability and its response to nutrient enrichment, as well as the effects of other global change drivers associated with altered biochemical cycles such as the increase in carbon dioxide and the acidification of marine ecosystems.

### Speculations

Our study provides a first step in the exploration of the consequences of stoichiometric constraints and temperature on ecological communities. It also calls for a better investigation of the factors determining the carrying capacity of biological systems in order to design mathematical models that can capture well community structure and dynamics. Is the carrying capacity of most ecological systems limited by nutrients or by other factors such as space or light? This is an important and, to our knowledge, unanswered question. Our study and others before us (Andersen et al. 2004) indicate that the popular Rosenzweig–MacArthur model is not appropriate to model nutrient limited systems as this model often violates the principle of mass conservation popularized by the French chemist Antoine Lavoisier with the famous quote “rien ne se perd, rien ne crée, tout se transforme” (‘nothing is lost, nothing creates, everything transforms’). The Rosenzweig– MacArthur model should be limited to systems where nutrients are not the main limit for carrying capacity. We speculate these biological systems are seldom: food quantity and quality are major determinants of growth, from cells to populations. This would call into question the numerous studies using the Rosenzweig–MacArthur model to assess to impacts of nutrient enrichment and other global change drivers on community dynamics and stability. We would thus need a different standard model that satisfies mass conservation and accounts for important nutrient feedbacks between consumers and resources. These nutrient-based models already exists (Daufresne and Loreau 2001, Andersen et al. 2004, Uszko et al. 2017, our study) and it is now time to use them in global change ecology, especially when investigating nutrient enrichment.

## Supplementary Material

Appendix

## Data Availability

R codes are available on Dryad Digital Repository (<https://doi.org/10.5061/dryad.msbcc2fv2>) (Sentis et al. 2021).
