# Supplementary material for: Stoichiometric constraints modulate temperature and nutrient effects on biomass distribution and community stability
Source: Oikos. Author manuscript; Available in PMC 2023 Jan 12. (PMC7614052; doi:10.1111/oik.08601)
Supplement: Appendix [file EMS159329-supplement-Appendix.docx]

**Supplementary information**

**Stoichiometric constraints modulate the effects of temperature and nutrients on biomass distribution and community stability**

**Text S1.** Derivation of the Stoichiometric Rosenzweig-MacArthur (SRM) model

The model studied in the main text is very similar to previous stoichiometric consumer-resource models (Andersen, 1997; Loladze *et al.*, 2000; Andersen *et al.*, 2004; Diehl *et al.*, 2005)[. To make our paper self-contained, we here present the model assumptions and derive the model equations (eqs. 3-6 in main text).](#_ENREF_18) Our objective was not to develop a complex and very realistic stoichiometric model that would include additional important abiotic and biotic features such as light intensity (Diehl, 2007) [or compensatory feeding](#_ENREF_17) (Cruz-Rivera & Hay, 2000)[. Instead, we aimed at introducing two fundamental stoichiometric features (i.e. stoichiometric flexibility and stoichiometric imbalance) and investigate how these stoichiometric considerations can change predictions of the Rosenzweig-MacArthur model. We assumed that resource and consumer production are limited by energy and a single mineral nutrient. Moreover, we assume the system is closed for nutrients. Thus, nutrient supply originates exclusively from excretion and remineralization of biomass. The total amount of nutrients in the system (](#_ENREF_15)*N*_tot_) is then a measure of nutrient enrichment. As elemental homeostasis is much stronger for consumers compared to primary producers (Andersen, 1997)[, we assumed the nutrient quota of the consumer](#_ENREF_4) *Q*_C_ to be constant whereas the nutrient quota of the resource *Q*_R_ is flexible. Four differential equations determine the dynamics of four state variables, that is, the concentrations of resource (*R*) and consumer (*C*) carbon biomasses and of dissolved mineral nutrients (*N*), and the nutrient quota of the resource (*Q*_R_):

$C=\left( e\frac{aR}{1+ahR}-m \right)C$ (S1)

$R=r\left( 1-\frac{Qmin}{Q_{R}} \right)R-\frac{aR}{1+ahR}C$ (S2)

$Q_{R}=h\left( N \right)-r\left( Q_{R}-Qmin \right)$ (S3)

$N=\left( Q_{R}-Q_{C}e \right)\frac{aR}{1+ahR}C+Q_{C}mC-h\left( N \right)R$ (S4)

As in the RM model, rates of change of the consumer and resource biomass densities $C$ and $R$ depend on their respective carbon biomass densities *C* and *R* (gC.m^-3^), except that the resource population growth rate follows the Droop equation (Droop, 1974) [and is now limited by its nutrient quota](#_ENREF_19) *Q*_R_ relative to the minimum nutrient quota *Q*_min_. Rate of change of *Q*_R_ depends on the nutrient uptake rate by the resource species *h*(*N*) and the amount of nutrient invested in growth (eqn S3). *h*(*N*) is the specific resource nutrient uptake rate and can be represented by a Michaelis-Menten model where the amount of nutrient uptake saturates at high nutrient concentrations.

With the mass-balance equation, we get that the total amount of nutrient is the sum of the free nutrient plus the nutrient fixed in the resource biomass plus the nutrient fixed in the consumer biomass: *N*_tot_ = *N* + *Q*_R_*R* + *Q*_C_*C.* As Eqns S1-S4 conserve total biomass (the system is closed), the time derivative of *N*_tot_ is zero. We can thus replace one of the four differential equations S1-S4 with the algebraic equation *N*_tot_ = *N* + *Q*_R_*R* + *Q*_C_*C*:

$C=\left( e\frac{aR}{1+ahR}-m \right)C$ (S5)

$R=r\left( 1-\frac{Qmin}{Q_{R}} \right)R-\frac{aR}{1+ahR}C$ (S6)

$N=\left( Q_{R}-Q_{C}e \right)\frac{aR}{1+ahR}C+Q_{C}mC-h\left( N \right)R$ (S7)

$N_{tot}={N+Q}_{R}R+Q_{C}C$ (S8)

It is possible to derive a simpler model by reducing the number of dimensions in the above model from three to two. This model reduction is based on the assumption that free nutrients are taken up very quickly relative to the dynamics of the consumer and resource biomasses. This corresponds to taking *h*(*N*) large, $h\left( N \right)=\frac{1}{\varepsilon}\tilde{h}\left( N \right)$ for small ε. The fast dynamics (on the timescale t ~ ε) are

$N=\frac{-1}{\varepsilon}\tilde{h}\left( N \right)R+\left( slowercontributions \right)$ (S9)

Which converge to N → 0, and $Q_{R}\to\frac{N_{tot}-Q_{C}C}{R}$ with *N*_tot_ the total nutrient in the system. In other words, *N* in dead and excreted matter is immediately recycled and acquired by the resource species. When substituting the quasi-steady-state in eqns. (S5, S6), we get the resulting dynamics (on the timescale t ~1):

$C=\left( e\frac{aR}{1+ahR}-m \right)C$ (S10)

$R=r\left( 1-\frac{Qmin}{Q_{R}} \right)R-\frac{aR}{1+ahR}C$ (S11)

$N_{tot}=Q_{R}R+Q_{C}C$ (S12)

From the nutrient conservation equation (eqn. S12) we obtain that $Q_{R}=\frac{N_{tot}-Q_{C}C}{R}$. The intuitive interpretation is that the resource nutrient quota *Q*_R_ decreases with the density of the resource population and with the density of nutrient stored in the consumer biomass. In contrast to eqns S5-S8, the reduced model has only two differential equations and one algebraic equation. It can be equivalently written as a set of three differential equations with $C$ and $R$ similar as equations S10 and S11 and with $Q_{R}=\frac{d\frac{N_{tot}-Q_{C}C}{R}}{dt}=\left( Q_{R}-Q_{C}e \right)\frac{aR}{1+ahR}C+\frac{Q_{C}mC}{R}-r\left( Q_{R}-Qmin \right).$

In the RM model, the growth rate of the consumer population is assumed to depend only on resource density. We relaxed this assumption by making the population growth rate of the consumer dependent on both the resource quality (i.e. nutrient quota) and quantity (i.e. density). In the SRM model, consumer production is also limited by resource quality as the consumer assimilation efficiency *e* is a saturating function of resource nutrient quota *Q*_R_:

$e\left( Q_{R} \right)=e_{max}\frac{Q_{R}}{Q_{R}+Q_{C}}$ (S13)

The intuitive interpretation of eqn. S13 is that resource quality is not a limiting factor for consumer growth as long as the nutrient content of the resource is superior to the nutrient content of the consumer (i.e. *Q*_R_ > *Q*_C_). In other words, when *Q*_R_ >> *Q*_C_, *e*(*Q*_R_) → *e*_max_ and when *Q*_R_ << *Q*_C_, *e*(*Q*_R_) → 0. By replacing *e* by *e*(*Q*_R_) in eqn. S10, we obtain the SRM model.

**Text S2. Differences in biomass ratios predicted by the two models**

Here we show that the equilibrium consumer-to-resource biomass ratio in the model with stoichiometric constraints (SRM model) is always smaller than the one in the model without stoichiometric constraints (RM model), keeping the same parameter values. For simplicity we assume for both models that the consumer and the resource persist at equilibrium, and we do not consider the stability of the equilibrium point (in particular, the equilibrium might be unstable at the center of a limit cycle). We indicate the equilibrium values of the non-stoichiometric model by the superscript “ns" and the equilibrium values of the stoichiometric model by the superscript “s". We use the same superscripts to distinguish the assimilation efficiencies of both models.

*Model without stoichiometric constraints*

The model is defined as

$R=r\left( 1-\frac{R}{K} \right)R-f\left( R \right)C$ (S14)

$C=\left( e^{ns}f\left( R \right)-m \right)C$ (S15)

With $f\left( R \right)=\frac{aR}{1+ahR}$ and *K* = *N*_tot_/*Q*_min_.

By solving equation (S15) we get the resource biomass at equilibrium:

$R^{ns}=\frac{1}{a\left( \frac{e^{ns}}{m}-h \right)}$ (S16)

From equation (S14) we get the consumer biomass at equilibrium. It follows from $f\left( R^{ns} \right)C^{ns}=r$ $(1-\frac{R^{ns}}{K})R^{ns}$ , or $C^{ns}=r$ $(1-\frac{R^{ns}}{K})\frac{R^{ns}}{f\left( R^{ns} \right)}$

Hence, the consumer-to-resource biomass ratio is

$\frac{C^{ns}}{R^{ns}}=\frac{re^{ns}}{m}$ $(1-\frac{R^{ns}}{K})=\frac{re^{ns}}{m}$ $(1-\frac{{Q_{min}R}^{ns}}{N_{tot}})$ (S17)

*Model with stoichiometric constraints*

The model is defined as

$R=r\left( 1-\frac{Q_{min}}{Q_{R}} \right)R-f\left( R \right)C$ (S18)

$C=\left( e^{s}\left( Q_{R} \right)f\left( R \right)-m \right)C$ (S19)

With $e^{s}\left( Q_{R} \right)=e_{max}\frac{Q_{R}}{Q_{C}+Q_{R}},f\left( R \right)=\frac{aR}{1+ahR}$ and $N_{tot}=Q_{R}R+Q_{C}C.$

From equation (S19) we have $e^{s}\left( Q_{R}^{s} \right)f\left( R^{s} \right)=m$, or

$R^{s}=\frac{1}{a\left( \frac{e^{s}\left( Q_{R}^{s} \right)}{m}-h \right)}$ (S20)

From equation (S18) we have from $f\left( R^{s} \right)C^{s}=r$ (1-$\frac{Q_{min}}{Q_{R}^{s}})R^{s}$, or

$C^{s}=\frac{e^{s}\left( Q_{R}^{s} \right)}{m}\left( 1-\frac{Q_{min}}{Q_{R}^{s}} \right)R^{s}$

Hence, the consumer-to-resource biomass ratio is

$\frac{C^{s}}{R^{s}}=\frac{e^{s}\left( Q_{R}^{s} \right)}{m}\left( 1-\frac{Q_{min}}{Q_{R}^{s}} \right)=\frac{e^{s}\left( Q_{R}^{s} \right)}{m}\left( 1-\frac{{R^{s}Q}_{min}}{N_{tot}-Q_{C}C^{s}} \right)$ (S21)

We now compare the biomass ratios of equations (S17) and (S21). We have *e*^ns^ = *e*_max_ as the RM model assumes that resource stoichiometry is not limiting and conversion efficiency is thus at its maximal value. However, conversion efficiency can be much lower when the resource is of poor quality (i.e. when there is a stoichiometric unbalance between the consumer and the resource nutrient: carbon ratio) (Elser *et al.*, 2000; Elser *et al.*, 2007)[. In other words, the consequence of stoichiometric constraints is to lower the values of conversion efficiency from the RM model. We thus obtain:](#_ENREF_21)

$e^{ns}\geq e^{s}\left( Q_{R}^{s} \right)$. (S22)

Using this inequality, we get $a\left( \frac{e^{ns}}{m}-h \right)\geq a\left( \frac{e^{s}\left( Q_{R}^{s} \right)}{m}-h \right)$, and by equations (S16) and (S20), we see that $R^{ns}\leq R^{s}$ (S23)

Clearly, we always have $N_{tot}\geq N_{tot}-Q_{C}C^{s}$. Combining this with equation (S23), we get $\frac{R^{ns}}{N_{tot}}\leq\frac{R^{s}}{N_{tot}-Q_{C}C^{s}}$ and

$1-$ $\frac{{Q_{min}R}^{ns}}{N_{tot}}\geq$ $1-\frac{Q_{min}R^{s}}{N_{tot}-Q_{C}C^{s}}$ (S24)

Finally, from equations (S22) and (S24),

$\frac{re^{ns}}{m}$ $(1-\frac{{Q_{min}R}^{ns}}{N_{tot}})\geq\frac{re^{s}\left( Q_{R}^{s} \right)}{m}\left( 1-\frac{Q_{min}R^{s}}{N_{tot}-Q_{C}C^{s}} \right),$ (S25)

showing that $\frac{C^{ns}}{R^{ns}}\geq\frac{C^{s}}{R^{s}}$.

**Table S1.** Definitions and units of model parameters, from Uszko *et al.* (2017) for the grazer *Daphnia hyalina* feeding on the green algae *Monoraphidium minutum*[.](#_ENREF_69) For temperature-dependent parameters, we list the value of the scaling constant *Q*_0_ (in units of the parameter) and the values of either the activation energy *E*_Q_ (eV, when temperature dependence is monotonous, eqn. 7) or of the temperature *T*_opt_ (Kelvin) at which the parameter value reaches a maximum/minimum and the width *s* (Kelvin) of this bell-/U-shaped function (when temperature dependence is non-monotonous, eqn. 8). Biomass and nutrients are expressed in units of carbon (C) and phosphorus (P), respectively

| Parameter | Temperature independent value  Thermal parameters | Unit | Definition | Reference |  |
| --- | --- | --- | --- | --- | --- |
| *r* | *r*_0_ = 2.2; *T*_opt_ = 298.15; *s* = 12.0 | 1/d | Intrinsic rate of resource net production (gross production – biosynthesis costs) | Uszko et al. 2017 |  |
| *h* | *h*_0_ = 0.17; *T*_opt_ = 294.1; *s* = 6.4 | d | Handling time | Uszko et al. 2017 |  |
| *a* | *a*_0_ = 8.9; *T*_opt_ = 296.0; *s* = 9.4 | m^3^/(gC d) | Attack rate | Uszko et al. 2017 |  |
| *m* | *m*_0_ = 4.4 × 10^8^; *E*_m_ = 0.55 | 1/d | Consumer mortality plus maintenance rate |  |  |
| *e*_max_ | 0.385 | - | Maximum assimilation efficiency | Peters 1983 |  |
| *Q*_C_ | 0.042 | g P/g C | Consumer P:C ratio | Diehl 2005 |  |
| *Q*_min_ | 0.009 | g P/g C | Minimum nutrient quota | Diehl 2005 |  |
| *Q*_R_ | Variable | g P/g C | Resource P:C ratio |  |  |
| N_tot_ | Variable | g P/m^3^ | Total nutrients in the system |  |  |
| T | Variable | K | Temperature |  |  |

**Fig. S1.** Thermal functions used to parametrize the model (adapted from Uszko et al. 2017)


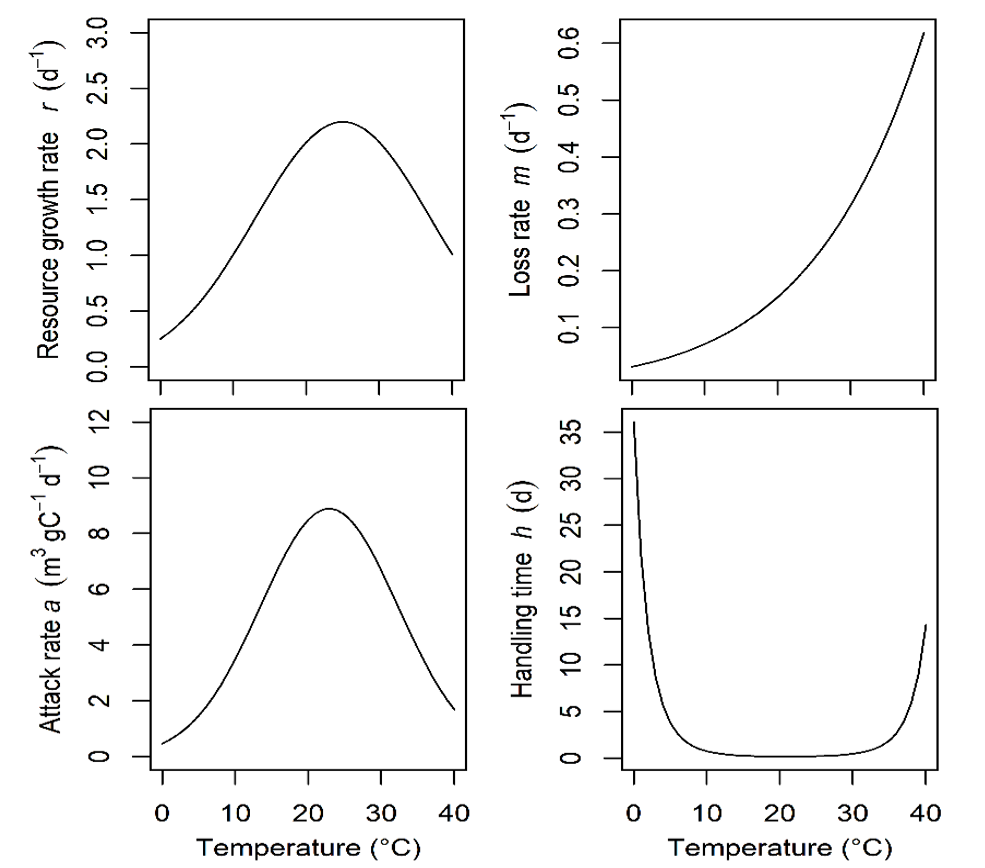


**Fig. S2.** Consumer-resource biomass ratio (log scale) along the temperature gradient for the Rosenzweig-MacArthur (RM, green lines) and the Stoichiometric Rosenzweig-MacArthur (SRM, black lines) models at three nutrient concentrations (0.008, 0.02, and 0.032 gP.m^-3^). In each panel, the dotted lines represent unstable solutions whereas full lines represent stable solutions. The thin horizontal dotted line represents biomass ratio of one; i.e. the biomass densities of the resource and the consumer are equal.


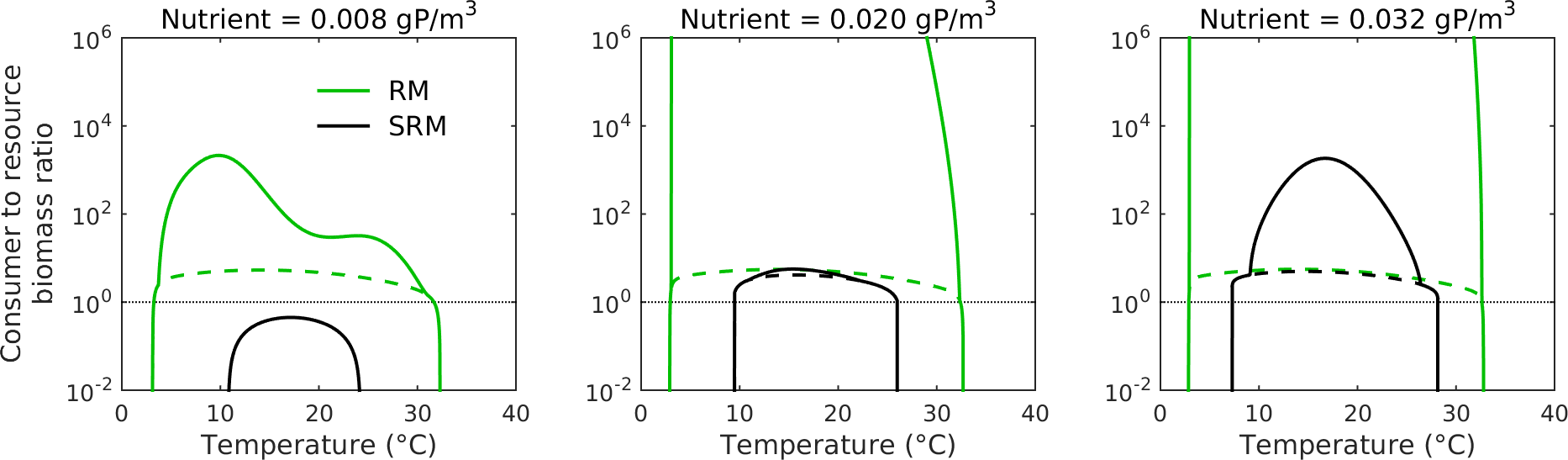


**Fig. S3**. Effective assimilation efficiency *e*_ef_ and carrying capacity *K*_ef_ from the Stoichiometric Rosenzweig-MacArthur (SRM) model along the temperature gradient at three nutrient levels (0.004, 0.015, and 0.032 gP.m^-3^) with *Q*_C_ = 0.042. Full lines represent temperature and nutrient scenarios for which both the resource and consumer persist whereas dotted lines represent scenarios for which only the resource persists. Effective assimilation efficiency was calculated as *e*_ef_ = *e*_max_*Q*_R_/(*Q*_R_+*Q*_C_), with *Q*_R_ the equilibrium solution of the SRM model and the effective carrying capacity as *K*_ef_ = *Q*_R_*R*/*Q*_min_ = (*N*_tot_-*Q*_C_*C*)/*Q_min_*, with *Q*_R_, *R* and *C* the equilibrium solutions of the SRM model.
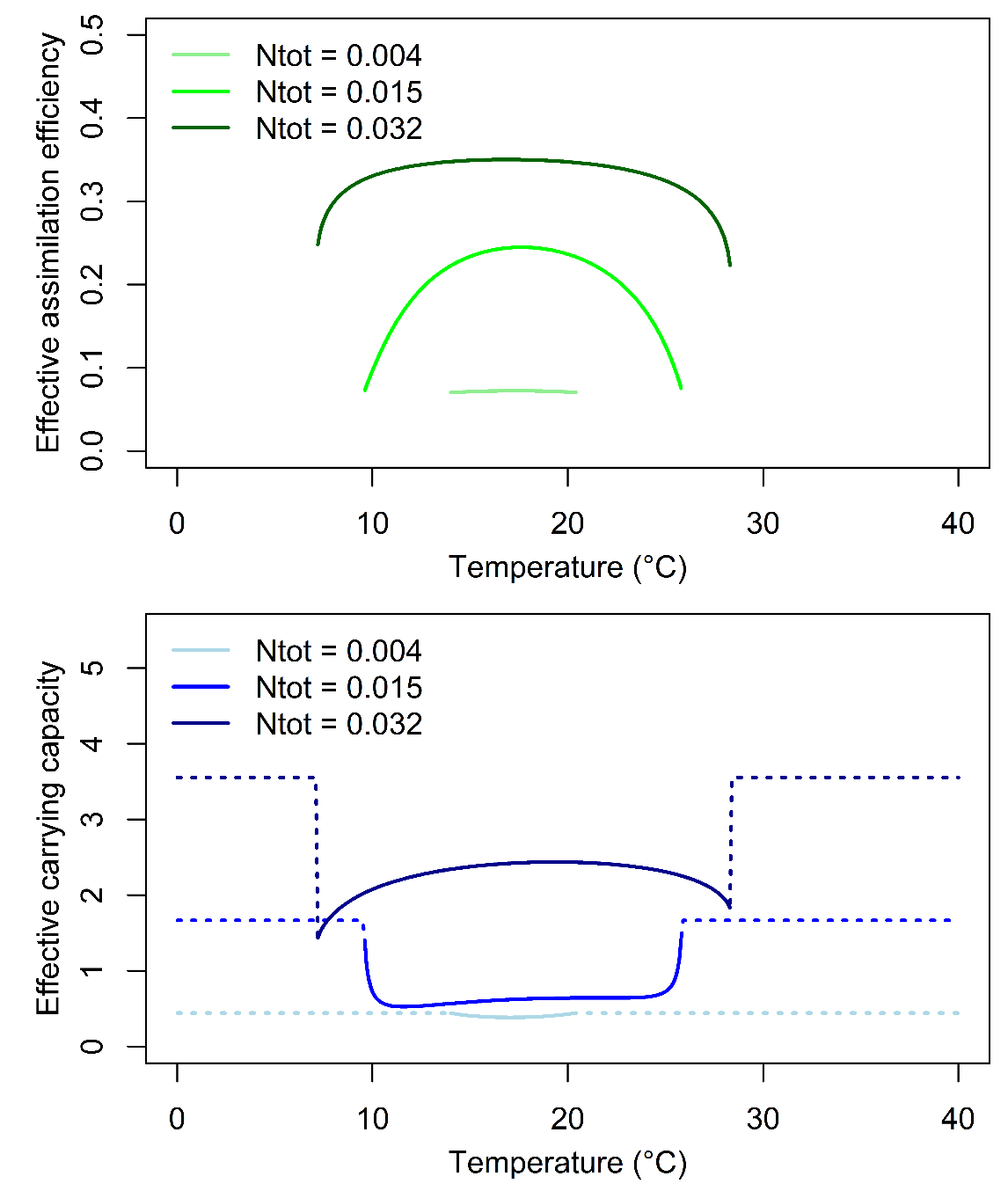


**References**

Ahlgren G (1987) Temperature functions in biology and their application to algal growth constants. *Oikos,* **49**, 177-190.

Amarasekare P (2015) Effects of temperature on consumer-resource interactions. *Journal of Animal Ecology,* **84**, 665-679.

Amarasekare P, Coutinho RM (2014) Effects of temperature on intraspecific competition in ectotherms. *The American Naturalist,* **184**, E50-E65.

Andersen T (1997) *Pelagic nutrient cycles: herbivores as sources and sinks,* Springer-Verlag, Berlin, Germany.

Andersen T, Elser JJ, Hessen DO (2004) Stoichiometry and population dynamics. *Ecology Letters,* **7**, 884-900.

Andersen T, Hessen DO (1991) Carbon, nitrogen, and phosphorus content of freshwater zooplankton. *Limnology and Oceanography,* **36**, 807-814.

Anneville O, Gammeter S, Straile D (2005) Phosphorus decrease and climate variability: mediators of synchrony in phytoplankton changes among European peri-alpine lakes. *Freshwater Biology,* **50**, 1731-1746.

Barbier M, Loreau M (2019) Pyramids and cascades: a synthesis of food chain functioning and stability. *Ecology Letters,* **22**, 405-419.

Bezemer TM, Jones TH (1998) Plant-insect herbivore interactions in elevated atmospheric C02: quantitative analyses and guild effects. *Oikos,* **82**, 212-222.

Binzer A, Guill C, Brose U, Rall BC (2012) The dynamics of food chains under climate change and nutrient enrichment. *Philosophical Transactions of the Royal Society B: Biological Sciences,* **367**, 2935-2944.

Binzer A, Guill C, Rall BC, Brose U (2016) Interactive effects of warming, eutrophication and size structure: impacts on biodiversity and food‐web structure. *Global Change Biology,* **22**, 220-227.

Boit A, Martinez ND, Williams RJ, Gaedke U (2012) Mechanistic theory and modelling of complex food-web dynamics in Lake Constance. *Ecology Letters,* **15**, 594-602.

Borer ET, Bracken ME, Seabloom EW *et al.* (2013) Global biogeography of autotroph chemistry: is insolation a driving force? *Oikos,* **122**, 1121-1130.

Brown JH, Gillooly JF, Allen AP, Savage VM, West GB (2004) Toward a metabolic theory of ecology. *Ecology,* **85**, 1771-1789.

Cherif, M. & Loreau, M. (2009). When microbes and consumers determine the limiting nutrient of autotrophs: a theoretical analysis. Proceedings of the Royal Society B: Biological Sciences, 276, 487-497.

Cherif, M. & Loreau, M. (2010). Towards a more biologically realistic use of Droop's equations to model growth under multiple nutrient limitation. Oikos, 119, 897-907.

Cruz-Rivera E, Hay ME (2000) Can quantity replace quality? Food choice, compensatory feeding, and fitness of marine mesograzers. *Ecology,* **81**, 201-219.

Daufresne, T. & Loreau, M. (2001). Plant–herbivore interactions and ecological stoichiometry: when do herbivores determine plant nutrient limitation? Ecology Letters, 4, 196-206.

Del Giorgio PA, Gasol JM (1995) Biomass Distribution in Freshwater Plankton Communities. *The American Naturalist,* **146**, 135-152.

Diehl S (2007) Paradoxes of enrichment: effects of increased light versus nutrient supply on pelagic producer-grazer systems. *The American Naturalist,* **169**, E173-E191.

Diehl S, Berger S, Wöhrl R (2005) Flexible nutrient stoichiometry mediates environmental influences on phytoplankton and its resources. *Ecology,* **86**, 2931-2945.

Droop M (1974) The nutrient status of algal cells in continuous culture. *Journal of the Marine Biological Association of the United Kingdom,* **54**, 825-855.

Elser J, Sterner R, Gorokhova Ea *et al.* (2000) Biological stoichiometry from genes to ecosystems. *Ecology Letters,* **3**, 540-550.

Elser JJ, Bracken ME, Cleland EE *et al.* (2007) Global analysis of nitrogen and phosphorus limitation of primary producers in freshwater, marine and terrestrial ecosystems. *Ecology Letters,* **10**, 1135-1142.

Elser JJ, Loladze I, Peace AL, Kuang Y (2012) Lotka re-loaded: modeling trophic interactions under stoichiometric constraints. *Ecological Modelling,* **245**, 3-11.

Elton C (1927) *Animal ecology,* Sidgwick & Jackson, LTD, London.

Englund G, Ohlund G, Hein CL, Diehl S (2011) Temperature dependence of the functional response. *Ecology Letters,* **14**, 914-921.

Enquist BJ, West GB, Charnov EL, Brown JH (1999) Allometric scaling of production and life-history variation in vascular plants. *Nature,* **401**, 907-911.

Falkowski PG, Barber RT, Smetacek V (1998) Biogeochemical controls and feedbacks on ocean primary production. *Science,* **281**, 200-206.

Finkel ZV, Beardall J, Flynn KJ, Quigg A, Rees TAV, Raven JA (2009) Phytoplankton in a changing world: cell size and elemental stoichiometry. *Journal of Plankton Research,* **32**, 119-137.

Fussmann KE, Schwarzmüller F, Brose U, Jousset A, Rall BC (2014) Ecological stability in response to warming. *Nature Climate Change,* **4**, 206-210.

Gilarranz LJ, Mora C, Bascompte J (2016) Anthropogenic effects are associated with a lower persistence of marine food webs. *Nature communications,* **7**, 10737.

Gilbert B, Tunney TD, McCann KS *et al.* (2014) A bioenergetic framework for the temperature dependence of trophic interactions. *Ecology Letters,* **17**, 902-9014.

Hessen DO, Ågren GI, Anderson TR, Elser JJ, De Ruiter PC (2004) Carbon sequestration in ecosystems: the role of stoichiometry. *Ecology,* **85**, 1179-1192.

Hessen DO, Færøvig PJ, Andersen T (2002) Light, nutrients, and P:C ratios in algae: grazer performance related to food quality and quantity. *Ecology,* **83**, 1886-1898.

Iles AC (2014) Towards predicting community level effects of climate: relative temperature scaling of metabolic and ingestion rates. *Ecology,* **95**, 2657–2668.

Irigoien X, Huisman J, Harris RP (2004) Global biodiversity patterns of marine phytoplankton and zooplankton. *Nature,* **429**, 863-867.

Jensen CX, Ginzburg LR (2005) Paradoxes or theoretical failures? The jury is still out. *Ecological Modelling,* **188**, 3-14.

Jonsson T (2017) Conditions for eltonian pyramids in Lotka-Volterra food chains. *Scientific reports,* **7**, 10912.

Kratina P, Greig HS, Thompson PL, Carvalho-Pereira TSA, Shurin JB (2012) Warming modifies trophic cascades and eutrophication in experimental freshwater communities. *Ecology,* **93**, 1421-1430.

Lindeman RL (1942) The trophic‐dynamic aspect of ecology. *Ecology,* **23**, 399-417.

Loladze I, Kuang Y, Elser JJ (2000) Stoichiometry in producer-grazer systems: linking energy flow with element cycling. *Bulletin of Mathematical Biology,* **62**, 1137-1162.

McAllister C, LeBrasseur R, Parsons T, Rosenzweig M (1972) Stability of enriched aquatic ecosystems. *Science,* **175**, 562-565.

McCauley DJ, Gellner G, Martinez ND *et al.* (2018) On the prevalence and dynamics of inverted trophic pyramids and otherwise top-heavy communities. *Ecology Letters,* **21**, 439-454.

McCauley E, Kalff J (1981) Empirical relationships between phytoplankton and zooplankton biomass in lakes. *Canadian Journal of Fisheries and Aquatic Sciences,* **38**, 458-463.

McCauley E, Nisbet RM, Murdoch WW, de Roos AM, Gurney WSC (1999) Large-amplitude cycles of *Daphnia* and its algal prey in enriched environments. *Nature,* **402**, 653-656.

Menge DNL, Hedin LO, Pacala SW (2012) Nitrogen and Phosphorus Limitation over Long-Term Ecosystem Development in Terrestrial Ecosystems. *PLoS ONE,* **7**, e42045.

Montoya JM, Raffaelli D (2010) Climate change, biotic interactions and ecosystem services. *Philosophical Transactions of the Royal Society B: Biological Sciences,* **365**, 2013-2018.

Nelson GC (2005) *Millennium ecosystem assessment: drivers of ecosystem change: summary chapter,* World Resources Institute, Washington, DC.

O'Connor MI, Piehler MF, Leech DM, Anton A, Bruno JF (2009) Warming and resource availability shift food web structure and metabolism. *PLoS Biology,* **7**, e1000178.

Petchey OL, McPhearson PT, Casey TM, Morin PJ (1999) Environmental warming alters food-web structure and ecosystem function. *Nature,* **402**, 69-72.

Peters RH (1983) *The ecological implications of body size,* Cambridge University Press, Cambridge.

R Development Core Team (2017) *R: a language and environment for statistical computing,* R Foundation for Statistical Computing, Vienna, Austria.

Rall BC, Brose U, Hartvig M, Kalinkat G, Schwarzmüller F, Vucic-Pestic O, Petchey OL (2012) Universal temperature and body-mass scaling of feeding rates. *Philosophical Transactions of the Royal Society B: Biological Sciences,* **367**, 2923-2934.

Rastetter EB, Ågren GI, Shaver GR (1997) Responses of N-limited ecosystems to increased CO_2_: a balanced-nutrition, coupled-element-cycles model. *Ecological Applications,* **7**, 444-460.

Rip JMK, McCann KS (2011) Cross-ecosystem differences in stability and the principle of energy flux. *Ecology Letters,* **14**, 733-740.

Robert W. Sterner, James J. Elser, Everett J. Fee, Stephanie J. Guildford, Thomas H. Chrzanowski (1997) The Light: Nutrient Ratio in Lakes: The Balance of Energy and Materials Affects Ecosystem Structure and Process. *The American Naturalist,* **150**, 663-684.

Rosenzweig ML (1971) Paradox of enrichment: destabilization of exploitation ecosystems in ecological time. *Science,* **171**, 385-387.

Sarmiento JL, Slater R, Barber R *et al.* (2004) Response of ocean ecosystems to climate warming. *Global Biogeochemical Cycles,* **18**, 1-23.

Sentis A, Binzer A, Boukal DS (2017) Temperature-size responses alter food chain persistence across environmental gradients. *Ecology Letters,* **20**, 852-862.

Sentis A, Hemptinne JL, Brodeur J (2012) Using functional response modeling to investigate the effect of temperature on predator feeding rate and energetic efficiency. *Oecologia,* **169**, 1117-1125.

Sentis A, Hemptinne JL, Brodeur J (2014) Towards a mechanistic understanding of temperature and enrichment effects on species interaction strength, omnivory and food-web structure. *Ecology Letters,* **17**, 785-793.

Soetaert K, Cash J, Mazzia F (2012) *Solving differential equations in R,* Springer Science & Business Media.

Sterner RW, Elser JJ (2002) *Ecological stoichiometry: the biology of elements from molecules to the biosphere,* Princeton University Press.

Sterner RW, Hessen DO (1994) Algal nutrient limitation and the nutrition of aquatic herbivores. *Annual Review of Ecology and Systematics,* **25**, 1-29.

Tabi A, Petchey OL, Pennekamp F (2019) Warming reduces the effects of enrichment on stability and functioning across levels of organisation in an aquatic microbial ecosystem. *Ecology Letters,* **22**, 1061-1071.

Thomas MK, Aranguren‐Gassis M, Kremer CT, Gould MR, Anderson K, Klausmeier CA, Litchman E (2017) Temperature–nutrient interactions exacerbate sensitivity to warming in phytoplankton. *Global Change Biology,* **23**, 3269-3280.

Thomas MK, Kremer CT, Klausmeier CA, Litchman E (2012) A global pattern of thermal adaptation in marine phytoplankton. *Science,* **338**, 1085-1088.

Tilman D (1982) *Resource competition and community structure,* Princeton university press.

Tranvik LJ, Downing JA, Cotner JB *et al.* (2009) Lakes and reservoirs as regulators of carbon cycling and climate. *Limnology and Oceanography,* **54**, 2298-2314.

Tylianakis JM, Didham RK, Bascompte J, Wardle DA (2008) Global change and species interactions in terrestrial ecosystems. *Ecology Letters,* **11**, 1351-1363.

Uszko W, Diehl S, Englund G, Amarasekare P (2017) Effects of warming on predator–prey interactions – a resource-based approach and a theoretical synthesis. *Ecology Letters,* **20**, 513-523.

Vasseur DA, McCann KS (2005) A mechanistic approach for modeling temperature-dependent consumer–resource dynamics. *American Naturalist,* **166**, 184-198.

Vucic-Pestic O, Ehnes RB, Rall BC, Brose U (2011) Warming up the system: higher predator feeding rates but lower energetic efficiencies. *Global Change Biology,* **17**, 1301-1310.

White T (1993) The inadequate environment. Nitrogen and the abundance of animals., Springer Verlag, Berlin.

Woods HA, Makino W, Cotner JB, Hobbie SE, Harrison JF, Acharya K, Elser JJ (2003) Temperature and the chemical composition of poikilothermic organisms. *Functional Ecology,* **17**, 237-245.

Yodzis P, Innes S (1992) Body size and consumer-resource dynamics. *The American Naturalist,* **139**, 1151-1175.

Yvon-Durocher G, Dossena M, Trimmer M, Woodward G, Allen AP (2015) Temperature and the biogeography of algal stoichiometry. *Global Ecology and Biogeography,* **24**, 562-570.
